# Evaluating Intervention Programs with a Pretest-Posttest Design: A Structural Equation Modeling Approach

**DOI:** 10.3389/fpsyg.2017.00223

**Published:** 2017-03-02

**Authors:** Guido Alessandri, Antonio Zuffianò, Enrico Perinelli

**Affiliations:** ^1^Department of Psychology, Sapienza University of RomeRome, Italy; ^2^Department of Psychology, Liverpool Hope UniversityLiverpool, UK

**Keywords:** experimental design, pretest-posttest, intervention, multiple group latent curve model, second order latent curve model, structural equation modeling, latent variables

## Abstract

A common situation in the evaluation of intervention programs is the researcher's possibility to rely on two waves of data only (i.e., pretest and posttest), which profoundly impacts on his/her choice about the possible statistical analyses to be conducted. Indeed, the evaluation of intervention programs based on a pretest-posttest design has been usually carried out by using classic statistical tests, such as family-wise ANOVA analyses, which are strongly limited by exclusively analyzing the intervention effects at the group level. In this article, we showed how second order multiple group latent curve modeling (SO-MG-LCM) could represent a useful methodological tool to have a more realistic and informative assessment of intervention programs with two waves of data. We offered a practical step-by-step guide to properly implement this methodology, and we outlined the advantages of the LCM approach over classic ANOVA analyses. Furthermore, we also provided a real-data example by re-analyzing the implementation of the Young Prosocial Animation, a universal intervention program aimed at promoting prosociality among youth. In conclusion, albeit there are previous studies that pointed to the usefulness of MG-LCM to evaluate intervention programs (Muthén and Curran, 1997; Curran and Muthén, 1999), no previous study showed that it is possible to use this approach even in pretest-posttest (i.e., with only two time points) designs. Given the advantages of latent variable analyses in examining differences in interindividual and intraindividual changes (McArdle, 2009), the methodological and substantive implications of our proposed approach are discussed.

## Introduction

Evaluating intervention programs is at the core of many educational and clinical psychologists' research agenda (Malti et al., 2016; Achenbach, 2017). From a methodological perspective, collecting data from several points in time (usually T ≥ 3) is important to test the long-term strength of intervention effects once the treatment is completed, such as in classic designs including pretest, posttest, and follow up assessments (Roberts and Ilardi, 2003). However, several factors could hinder the researcher's capacity to collect data at follow-up assessments, in particular the lack of funds, participants' poor level of monitoring compliance, participants' relocation in different areas, etc. Accordingly, the use of the less advantageous pretest-posttest design (i.e., before and after the intervention) often represents a widely used methodological choice in the psychological intervention field. Indeed, from a literature research on the database PsycINFO using the following string “*intervention* AND *pretest* AND *posttest* AND *follow-up*” limited to abstract section and with a publication date from January 2006 to December 2016, we obtained 260 documents. When we changed “AND *follow-up*” with “NOT *follow-up*” the results were 1,544 (see Appendix A to replicate these literature search strategies).

A further matter of concern arises from the statistical approaches commonly used for evaluating intervention programs in pretest-posttest design, mostly ANOVA-family analyses, which heavily rely on statistical assumptions (e.g., normality, homogeneity of variance, independence of observations, absence of measurement error, and so on) rarely met in psychological research (Schmider et al., 2010; Nimon, 2012).

However, all is not lost and some analytical tools are available to help researchers better assess the efficacy of programs based on a pretest-posttest design (see McArdle, 2009). The goal of this article is to offer a formal presentation of a latent curve model approach (LCM; Muthén and Curran, 1997) to analyze intervention effects with only two waves of data. After a brief overview of the advantageous of the LCM framework over classic ANOVA analyses, a step-by-step application of the LCM on real pretest-posttest intervention data is provided.

## Evaluation approaches: observed variables vs. latent variables

Broadly speaking, approaches to intervention evaluation can be distinguished into two categories: (1) approaches using *observed variables* and (2) approaches using *latent variables*. The first category includes widely used parametric tests such as Student's *t*, repeated measures analysis of variance (RM-ANOVA), analysis of covariance (ANCOVA), and ordinary least-squares regression (see Tabachnick and Fidell, 2013). However, despite their broad use, observed variable approaches suffer from several limitations, many of them ingenerated by the strong underlying statistical assumptions that must be satisfied. A first series of assumption underlying classic parametric tests is that the data being analyzed are normally distributed and have equal population variances (also called homogeneity of variance or *homoscedasticity* assumption). Normality assumption is not always met in real data, especially when the variables targeted by the treatment program are infrequent behaviors (i.e., externalizing conducts) or clinical syndromes (Micceri, 1989). Likewise, homoschedasticy assumption is rarely met in randomized control trial as a result of the experimental variable causing differences in variability between groups (Grissom and Kim, 2012). Violation of normality and homoscedasticity assumptions can compromise the results of classic parametric tests, in particular on rates of Type-I (Tabachnick and Fidell, 2013) and Type-II error (Wilcox, 1998). Furthermore, the inability to deal with measurement error can also lower the accuracy of inferences based on regression and ANOVA-family techniques which assume that the variables are measured without errors. However, the presence of some degree of measurement error is a common situation in psychological research where the focus is often on not directly observable constructs such as depression, self-esteem, or intelligence. Finally, observed variable approaches assume (without testing it) that the measurement structure of the construct under investigation is invariant across groups and/or time (Meredith and Teresi, 2006; Millsap, 2011). Thus, lack of satisfied statistical assumptions and/or uncontrolled unreliability can lead to the under or overestimation of the true relations among the constructs analyzed (for a detailed discussion of these issues, see Cole and Preacher, 2014).

On the other side, latent variable approaches refer to the class of techniques termed under the label structural equation modeling (SEM; Bollen, 1989) such as confirmatory factor analysis (CFA; Brown, 2015) and mean and covariance structures analysis (MACS; Little, 1997). Although a complete overview of the benefits of SEM is beyond the scope of the present work (for a thorough discussion, see Little, 2013; Kline, 2016), it is worthwhile mentioning here those advantages that directly relate to the evaluation of intervention programs. First, SEM can easily accommodate the lack of normality in the data. Indeed, several estimation methods with standard errors robust to non-normal data are available and easy-to-use in many popular statistical programs (e.g., MLM, MLR, WLSMV, etc. in M*plus*; Muthén and Muthén, 1998–2012). Second, SEM explicitly accounts for measurement error by separating the common variance among the indicators of a given construct (i.e., the latent variable) from their residual variances (which include both measurement error and unique sources of variability). Third, if multiple items from a scale are used to assess a construct, SEM allows the researcher to evaluate to what extent the measurement structure (i.e., factor loadings, item intercepts, residual variances, etc.) of such scale is equivalent across groups (e.g., intervention group vs. control group) and/or over time (i.e., pretest and posttest); this issue is known as measurement invariance (MI) and, despite its crucial importance for properly interpreting psychological findings, is rarely tested in psychological research (for an overview see Millsap, 2011; Brown, 2015). Finally, different competitive SEMs can be evaluated and compared according to their goodness of fit (Kline, 2016). Many SEM programs, indeed, print in their output a series of fit indexes that help the researcher assess whether the hypothesized model is consistent with the data or not. In sum, when multiple indicators of the constructs of interest are available (e.g., multiple items from one scale, different informants, multiple methods, etc.), latent variables approaches offer many advantages and, therefore, they should be preferred over manifest variable approaches (Little et al., 2009). Moreover, when a construct is measured using a single psychometric measure, there are still ways to incorporate the individuals' scores in the analyses as latent variables, and thus reduce the impact of measurement unreliability (Cole and Preacher, 2014).

## Latent curve models

Among latent variable models of change, latent curve models (LCMs; Meredith and Tisak, 1990), represent a useful and versatile tool to model stability and change in the outcomes targeted by an intervention program (Muthén and Curran, 1997; Curran and Muthén, 1999). Specifically, in LCM individual differences in the rate of change can be flexibly modeled through the use of two *continuous random latent variables*: The intercept (which usually represents the level of the outcome of interest at the pretest) and the slope (i.e., the mean-level change over time from the pretest to the posttest). In detail, both the intercept and the slope have a mean (i.e., the average initial level and the average rate of change, respectively) and a variance (i.e., the amount of inter-individual variability around the average initial level and the average rate of change). Importantly, if both the mean and the variance of the latent slope of the outcome *y* in the intervention group are statistically significant (whereas they are not significant in the control group), that means that there was not only an average effect of the intervention, but also some participants were differently affected by the program (Muthén and Curran, 1997). Hence, the assumption that participants respond to the treatment in the same way (as in ANOVA-family analyses) can be easily relaxed in LCM. Indeed, although individual differences may also be present in the ANOVA design, change occurs at the group level and, therefore, everyone is impacted in the same fashion after the exposure to the treatment condition.

As discussed by Muthén and Curran (1997), the LCM approach is particular useful for evaluating intervention effects when it is conducted within a multiple group framework (i.e., MG-LCM), namely when the intercept and the slope of the outcome of interest are simultaneously estimated in the intervention and control group. Indeed, as illustrate in our example, the MG-LCM allows the research to test if both the mean and the variability of the outcome *y* at the pretest are similar across intervention and control groups, as well as if the mean rate of change and its inter-individual variability are similar between the two groups. Therefore, the MG-LCM provides information about the efficacy of an intervention program in terms of both (1) its average (i.e., group-level) effect and (2) participants' sensitivity to differently respond to the treatment condition.

However, a standard MG-LCM cannot be empirically identified with two waves of data (Bollen and Curran, 2006). Yet, the use of multiple indicators (at least 2) for each construct of interest could represent a possible solution to overcome this problem by allowing the estimation of the intercept and slope as second-order latent variables (McArdle, 2009; Geiser et al., 2013; Bishop et al., 2015). Interestingly, although second-order LCMs are becoming increasingly common in psychological research due to their higher statistical power to detect changes over time in the variables of interest (Geiser et al., 2013), their use in the evaluation of intervention programs is still less frequent. In the next section, we present a formal overview of a second-order MG-LCM approach, we describe the possible models of change that can be tested to assess intervention effects in pretest-posttest design, and we show an application of the model to real data.

## Identification of a two-time point latent curve model using parallel indicators

**Figure 1:**
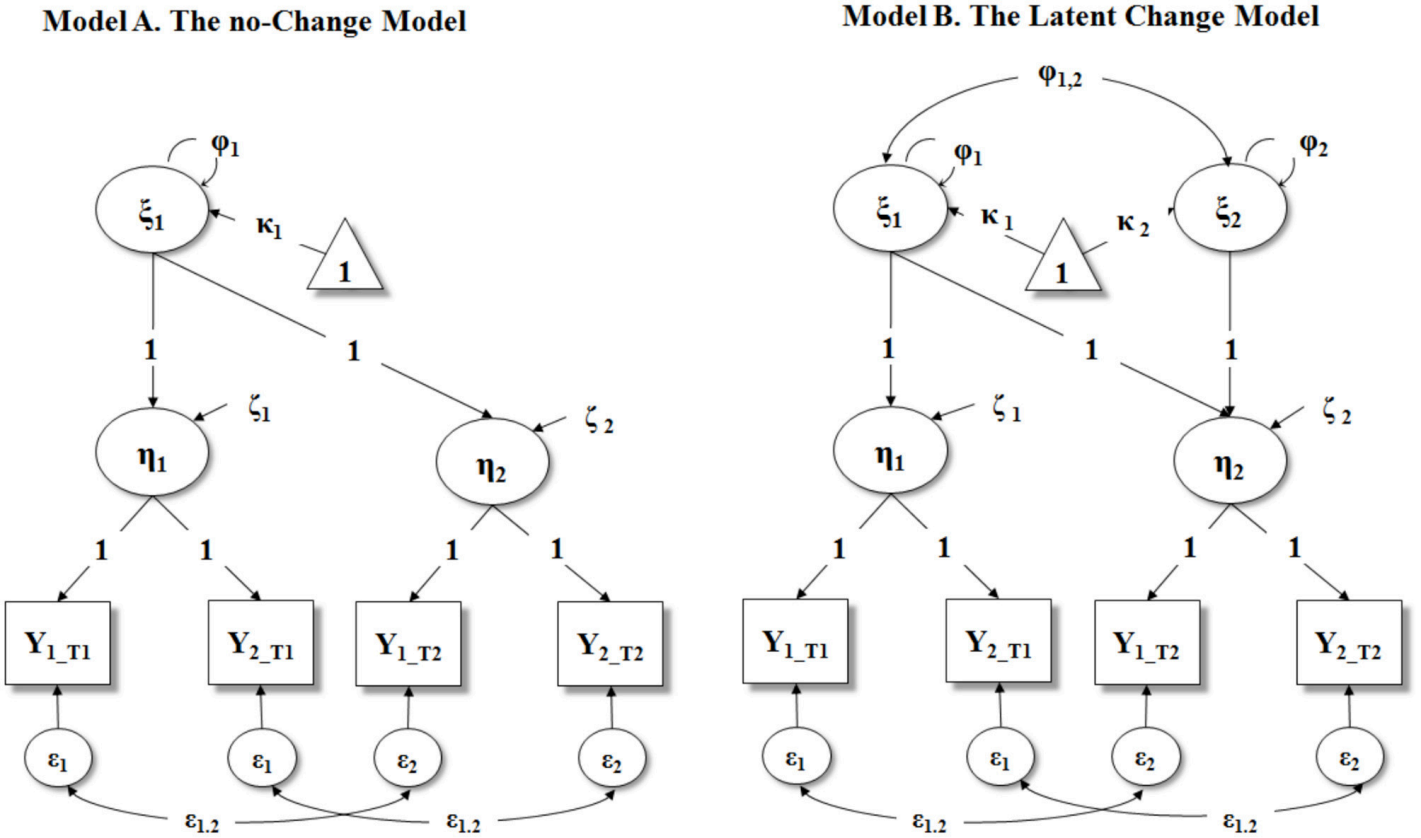
**Second Order Latent Curve Models with parallel indicators (i.e., residual variances of observed indicators are equal within the same latent variable: ε_1_ within η_1_and ε_2_ within η_2_)**. All the intercepts of the observed indicators (Y) and endogenous latent variables (η) are fixed to 0 (not reported in figure). In model A, the residual variances of η_1_ and η_2_ (ζ_1_ and ζ_2_, respectively) are freely estimated, whereas in Model B they are fixed to 0. ξ_1_, intercept; ξ_2_, slope; κ_1_, mean of intercept; κ_2_, mean of slope; ϕ_1_, variance of intercept; ϕ_2_, variance of slope; ϕ_12_, covariance between intercept and slope; η_1_, latent variable at T1; η_2_, latent variable at T2; Y, observed indicator of η; ε, residual variance/covariance of observed indicators.

When only two points in time are available, it is possible to estimate two LCMs: A No-Change Model (see Figure 1 Panel A) and a Latent Change Model (see Figure 1 Panel B). In the following, we described in details the statistical underpinnings of both these models.

### Latent change model

A *two-time points latent change model* implies two latent means (κ^k^), two latent factor variances (ζ^k^), plus the covariance between the intercept and slope factor (Φ^k^). This results in a total of 5+T model parameters, where T are the error variances for (y^k^) when allowing *VAR*(∈^k^) to change over time. In the case of a two waves of data (i.e., T = 2), this latent change model has 7 parameters to estimate from a total of (2) (3)/2+2 = 5 identified means, variances, and covariances of the observed variables. Hence, two waves of data are insufficient to estimate this model. However, this latent change model can be just-identified (i.e., zero degrees of freedom [df]) by constraining the residual variances of the observed variables to be 0. This last constraint should be considered structural and thus included in all two-time points latent change model. In this latter case, the variances of the latent variables (i.e., the latent intercept representing the starting level, and the latent change score) are equivalent to those of the observed variables. Thus, when fallible variables are used, this impedes to separate true scores from their error/residual terms.

A possible way to allow this latent change model to be over-identified (i.e., df ≥ 1) is by assuming the availability of at least two observed indicators of the construct of interest at each time point (i.e., T1 and T2). Possible examples include the presence of two informants rating the same behavior (e.g., caregivers and teachers), two scales assessing the same construct, etc. However, even if the construct of interest is assessed by only one single scale, it should be noted that psychological instruments are often composed by several items. Hence, as noted by Steyer et al. (1997), it is possible to randomly partitioning the items composing the scale into two (or more) parcels that can be treated as parallel forms. By imposing appropriate constraints on the loadings (i.e., λ^*k*^ = 1), the intercepts (τ^k^ = 0), within factor residuals (ε^k^ = ε), and by fixing to 0 the residual variances of the first-order latent variables η^k^ (ζ^k^ = 0), the model can be specified as a first-order measurement model plus a second-order latent change model (see Figure 1 Panel B). Given previous constraints of loadings, intercepts, and first order factor residual variances, this model is over-identified because we have (4) (5)/2+4 = 14 observed variances, covariances, and means. Of course, when three or more indicators are available, identification issues cease to be a problem. In this paper, we restricted our attention to the two parallel indicators case to address the more basic situation that a researcher can encounter in the evaluation of a two time-point intervention. Yet, our procedure can be easily extended to cases in which three or more indicators are available at each time point.

#### Specification

More formally, and under usual assumptions (Meredith and Tisak, 1990), the measurement model for the above two times latent change model in group *k* becomes:

(1) $$\begin{array}{l} {\text{y}}^{\text{k}}=\tau_{\text{y}}^{\text{k}}+\Lambda_{\text{y}}^{\text{k}}\;\eta^{\text{k}}+\in^{\text{k}}, \end{array}$$

where y^k^ is a *mp x 1* random vector that contains the observed scores, $\left\{y_{it}^{k}\right\}$, for the *ith* variable at time *t*, i ∈ {1,2,., p}, and t ∈ {1,2,., m}. The intercepts are contained in the *mp x 1* vector $\tau_{\text{y}}^{\text{k}}$, $\Lambda_{\text{y}}^{\text{k}}$ is a *mp x mq* matrix of factor loadings, η^k^ is a *mq x 1* vector of factor scores, and the unobserved error random vectors ∈^k^ is a *mp x 1* vector. The population vector mean, $\mu_{\text{y}}^{\text{k}}$, and covariance matrix, $\overset{\text{k}}{\underset{\text{y}}{\sum }}$, or Means and Covariance Structure (MACS) are:

(2) $$\begin{array}{r} \mu_{\text{y}}^{\text{k}}=\tau_{\text{y}}^{\text{k}}+\Lambda_{\text{y}}^{\text{k}}{\mu_{\eta }}^{\text{k}}\text{ and }\sum_{\text{y}}^{\text{k}}=\Lambda_{\text{y}}^{\text{k}}\sum_{\eta }^{\text{k}}\;\Lambda_{\text{y}}^{{\text{k}}^{\text{′}}}\;+\;\theta_{\varepsilon }^{\text{k}}, \end{array}$$

where $\mu_{\eta }^{\text{k}}$ is a vector of latent factors means, $\overset{\text{k}}{\underset{\eta }{\sum }}$ is the modeled covariance matrix, and $\theta_{\varepsilon }^{\text{k}}$ is a *mp* × *mp* matrix of observed variable residual covariances. For each column, fixing an element of $\Lambda_{\text{y}}^{\text{k}}$ to 1, and an element of $\tau_{\text{y}}^{\text{k}}$ to 0, identifies the model. By imposing increasingly restrictive constraints on elements of matrix Λ_y_ and τ_y_, the above two-indicator two-time points model can be identified.

The general equations for the structural part of a second order (SO) multiple group (MG) model are:

(3) $$\begin{array}{r} \eta^{\text{k}}=\Gamma^{\text{k}}\;\xi^{\text{k}}\;+\;\zeta^{\text{k}}, \end{array}$$

where Γ^k^ is a *mp x qr* matrix containing second order factor coefficients, ξ^k^ is a *qr* × *1* vector of second-order latent variables, and ζ^k^ is a *mq x 1* vector containing latent variable disturbance scores. Note that *q* is the number of latent factors and that *r* is the number of latent curves for each latent factor.

The population mean vector, $\mu_{\eta }^{\text{k}}$, and covariance matrix, $\overset{\text{k}}{\underset{\eta }{\sum }}$, based on (3) are

(4) $$\begin{array}{r} \mu_{\eta }^{\text{k}}\;=\;\Gamma^{\text{k}}\kappa^{\text{k}}\text{ and }\sum_{\eta }^{\text{k}}\;=\;\Gamma^{\text{k}}\Phi^{\text{k}}\Gamma^{{\text{k}}^{\text{′}}}+\psi^{\text{k}}, \end{array}$$

where Φ^k^ is a *r x r* covariance of the latent variables, and Ψ^k^ is a *mq* × *mq* latent variable residual covariance matrix. In the current application, what makes the difference in two models is the way in which matrices Γ^k^ and Φ^k^ are specified.

### Application of the SO-MG-LCM to intervention studies using a pretest-posttest design

The application of the above two-times LCM to the evaluation of an intervention is straightforward. Usually, in intervention studies, individuals are randomly assigned to two different groups. The first group (**G**_1_) is exposed to an intervention that takes place somewhere after the initial time point. The second group (**G**_2_), also called the control group, does not receive any direct experimental manipulation. In light of the random assignment, **G**_1_ and **G**_2_ can be viewed as two equivalent groups drawn by the same population and the effect of the intervention may be ascertained by comparing individuals' changes from T1 to T2 across these two groups.

Following Muthén and Curran (1997), an intercept factor should be modeled in both groups. However, only in the intervention group an additional latent change factor should be added. This factor is aimed at capturing the degree of change that is specific to the treatment group. Whereas, the absolute value for the latent mean of this factor can be interpreted as the change determined by the intervention in the intervention group, a significant variance indicates a meaningful heterogeneity in responding to the treatment. In this model $\alpha_{\text{y}}^{\text{k}}$ is a vector containing freely estimating mean values for the intercept (i.e., ξ^1^), and the slope (i.e., ξ^2^). $\Gamma_{\text{y}}^{\text{k}}$ is thus a *2 x 2* matrix, containing basis coefficients, determined in $\left[\begin{array}{l} 1\\ 1 \end{array}\right]$ for the intercept (i.e., ξ^1^) and $\left[\begin{array}{l} 0\\ 1 \end{array}\right]$ for the slope (i.e., ξ^2^). Φ^k^ is a *2 x 2* matrix containing variances and covariance for the two latent factors representing the intercept and the slope.

Given randomization, restricting the parameters of the intercept to be equal across the control and treatment populations is warranted in a randomized intervention study. Yet, baseline differences can be introduced in field studies where randomization is not possible or, simply, the randomization failed during the course of the study (Cook and Campbell, 1979). In such cases, the equality constraints related to the mean or to the variance of the intercept can be relaxed.

The influence of participants' initial status on the effect of the treatment in the intervention group can also be incorporated in the model (Cronbach and Snow, 1977; Muthén and Curran, 1997; Curran and Muthén, 1999) by regressing the latent change factor onto the intercept factor, so that the mean and variance of the latent change factor in the intervention group are expressed as a function of the initial status. Accordingly, this analysis captures to what extent inter-individual initial differences on the targeted outcome can predispose participants to differently respond to the treatment delivered.

### Sequence of models

We suggest a four-step approach to intervention evaluation. By comparing the relative fit of each model, researchers can have important information to assess the efficacy of their intervention.

#### Model 1: no-change model

A no-change model is specified for both intervention group (henceforth G1) and for control group (henceforth G2). As a first step, indeed, a researcher may assume that the intervention has not produced any meaningful effect, and therefore a no-change model (or strict stability model) should be simultaneously estimated in both the intervention and control group. In its more general version, the no-change model includes only a second-order intercept factor which represents the participants' initial level. Importantly, both the mean and variance of the second-order intercept factor are freely estimated across groups (see Figure 1 Panel A). More formally, in this model, Φ^k^ is a *qr x qr* covariance matrix of the latent variables, and Γ^k^ is a *mq x qr* matrix, containing for each latent variable, a set of basis coefficients for the latent curves.

#### Model 2: latent change model in the intervention group

In this model, a slope growth factor is estimated in the intervention group only. As previously detailed, this additional latent factor is aimed at capturing any possible change in the intervention group. According to our premises, this model represents the “target” model, attesting a significant intervention effect in G1 but not in G2. Model 1 is then compared with Model 2 and changes in fit indexes between the two models are used to evaluate the need of this further latent factor (see section Statistical Analysis).

#### Model 3: latent change model in both the intervention and control group

In model 3, a latent change model is estimated simultaneously in both G1 and G2. The fit of Model 2 is compared with the fit of Model 3 and changes in fit indexes between the two models are used to evaluate the need of this further latent factor in the control group. From a conceptual point of view, the goal of Model 3 is twofold because it allows the researcher: (a) to rule out the eventuality of “contaminations effects” between the intervention and control group (Cook and Campbell, 1979); (b) to assess a possible, normative mean-level change in the control group (i.e., a change that cannot be attributed to the treatment delivered). In reference to (b), indeed, it should be noted that some variables may show a normative developmental increase during the period of the intervention. For instance, a consistent part of the literature has identified an overall increase in empathic capacities during early childhood (for an overview, see Eisenberg et al., 2015). Hence, researchers aimed at increasing empathy-related responding in young children may find that both the intervention and control group actually improved in their empathic response. In this situation, both the mean and variance of the latent slope should be constrained to equality across groups to mitigate the risk of confounding intervention effects with the normative development of the construct (for an alternative approach when more than two time points are available, see Muthén and Curran, 1997; Curran and Muthén, 1999). Importantly, the tenability of these constraints can be easily tested through a delta chi square test (Δχ^2^) between the chi squares of the constrained model *vs*. unconstrained model. A significant Δχ^2^ (usually *p* < 0.05) indicates that the two models are not statistically equivalent, and the unconstrained model should be preferred. On the contrary, a non-significant Δχ^2^ (usually *p* > 0.05) indicates that the two models are statistically equivalent, and the constrained model (i.e., the more parsimonious model) should be preferred.

#### Model 4: sensitivity model

After having identified the best fitting model, the parameters of the intercept (i.e., mean and variance) should be constrained to equality across groups. This sensitivity analysis is crucial to ensure that both groups started with an equivalent initial status on the targeted behavior which is an important assumption in intervention programs. In line with previous analyses, the plausibility of initial status can be easily tested through the Δχ^2^ test. Indeed, given randomization, it seems likely to assume that participants in both groups are characterized by similar or identical starting levels, and the groups have the same variability. These assumptions lead to a *constrained* no-change no-group difference model. This model is the same as the previous one, except that κ^k^ = κ, or in our situation κ^1^ = κ^2^. Moreover, in our situation, *r* = *1, q* = *1, m* = *2*, and hence, Φ^k^ = Φ is a scalar, Γ^k^ = 1_2_, and Ψ^k^ = ΨI_2_ for each of the k^th^ population.

In the next section, the above sequence of models has been applied to the evaluation of a universal intervention program aimed to improve students' prosociality. We presented results from every step implied by the above methodology, and we offered a set of M*plus* syntaxes to allow researchers estimate the above models in their dataset.

## The young prosocial animation program

The Young Prosocial Animation (YPA; Zuffianò et al., 2012) is a *universal* intervention program (Greenberg et al., 2001) to sensitize adolescents to prosocial and empathic values (Zuffianò et al., 2012).

In detail, the YPA tries to valorize: (a) the status of people who behave prosocially, (b) the similarity between the “model” and the participants, and (c) the outcomes related to prosocial actions. Following Bandura's (1977) concept of *modeling*, in fact, people are more likely to engage in those behaviors they *value* and if the model is perceived as *similar* and with an *admired status*. The main idea is that valuing these three aspects could foster a *prosocial sensitization* among the participants (Zuffianò et al., 2012). In other terms, the goal is to promote the cognitive and emotional aspects of prosociality, in order to strengthen attitudes to act and think in a “prosocial way.” The expected change, therefore, is at the level of the personal dispositions in terms of an increased receptiveness and propensity for *prosocial thinking* (i.e., both the ability to take the point of view and to be empathetic rather than directly affecting the behaviors acted out by the individuals, as well as the ability to produce ideas and solutions that can help other people; Zuffianò et al., 2012). Due to its characteristics, YPA can be conceived as a first phase of *prosocial sensitization* on which implementing programs more appropriately direct to increase prosocial behavior (e.g., CEPIDEA program; Caprara et al., 2014). YPA aims to achieve this goal through a guided discussion following the viewing of some prosocial scenes selected from the film “Pay It Forward”^1^. After viewing each scene, a trained researcher, using a standard protocol guides a discussion among the participants highlighting: (i) the type of prosocial action (e.g., consoling, helping, etc.); (ii) the benefits for the actor and the target of the prosocial action; (iii) possible benefits of the prosocial action extended to the context (e.g., other persons, the more broad community, etc.); (iv) requirements of the actor to behave prosocially (e.g., being empathetic, bravery, etc.); (v) the similarity between the participant and the actor of the prosocial behavior; (vi) the thoughts and the feelings experienced during the viewing of the scene. The researcher has to complete the intervention within 12 sessions (1 h per session, once a week).

For didactic purposes, in the present study we re-analyzed data from an implementation of the YPA in three schools located in a small city in the South of Italy (see Zuffianò et al., 2012 for details).

### Hypotheses

We expected Model 2 (a latent change model in the intervention group and a no-change model in the control group) to be the best fitting model. Indeed, from a developmental point of view, we had no reason to expect adolescents showing a normative change in prosociality after such a short period of time (Eisenberg et al., 2015). In line with the goal of the YPA, we hypothesized an small-medium increase in prosociality in the intervention group. We also expected that both groups did not differ at T1 in absolute level of prosocial behaviors, ensuring that both intervention and control group were equivalent. Finally, we explored the influence of participants' initial status on the treatment effect, a scenario in which those participants with lower initial level of prosociality benefitted more from attending the YPA session.

## Methods

### Design

The study followed a *quasi-experimental design*, with both the intervention and control groups assessed at two different time points: Before (Time 1) YPA intervention and 6 months after (Time 2). Twelve classrooms from three schools (one middle school and two high schools) participated in the study during the school year 2008–2009. Each school has ensured the participation of 4 classes that were randomly assigned to intervention and control group (two classes to intervention group and two classes to control group).^2^ In total, six classes were part of intervention group and six classes of control group. The students from the middle school were in the eighth grade (third year of secondary school in Italy), whereas the students from the two high schools were in the ninth (first year of high school in Italy) and tenth grade (second year of high school in Italy).

### Participants

The YPA program was implemented in a city in the South of Italy. A total amount of 250 students participated in the study: 137 students (51.8% males) were assigned to the intervention group and 113 (54% males) to the control group. At T2 students were 113 in the intervention group (retention rate = 82.5%) and 91 in the control group (retention rate = 80.5%). Little's test of missingness at random showed a non-significant chi-squared value [$\chi_{\left(2\right)}^{2}$ = 4.698, *p* = 0.10]; this means that missingness at posttest is not affected by the levels of prosociality at pretest. The mean age was 14.2 (*SD* = 1.09) in intervention group, and 15.2 (*SD* = 1.76) in control group. Considering socioeconomic status, the 56.8% of families in intervention group and the 60.0% in control group were one-income families. The professions mostly represented in the two groups were the “worker” among the fathers (the 36.4% in intervention group and the 27.9% in control group) and the “housewife” among the mothers (the 56.0% in the intervention group and the 55.2% in the control group). Parent's school level was approximately the same between the two groups: Most of parents in the intervention group (43.5%) and in the control group (44.7%) had a middle school degree.

### Measures

#### Prosociality

Participants rated their prosociality on a 16-item scale (5-point Likert scale: 1 = *never/almost never true*; 5 = *almost always/always true*) that assesses the degree of engagement in actions aimed at sharing, helping, taking care of others' needs, and empathizing with their feelings (e.g., “*I try to help others*” and “*I try to console people who are sad*”). The alpha reliability coefficient was 0.88 at T1 and 0.87 at T2. The scale has been validated on a large sample of respondents (Caprara et al., 2005) and has been found to moderately correlate (*r* > 0.50) with other-ratings of prosociality (Caprara et al., 2012).

### Statistical analysis

All the preceding models were estimated by maximum likelihood (ML) using M*plus* program 7 (Muthén and Muthén, 1998–2012). Missing data were handled using full information maximum likelihood (FIML) estimation, which draws on all available data to estimate model parameters without imputing missing values (Enders, 2010). To evaluate the goodness of fit, we relied on different criteria. First we evaluated the values assumed by the χ^2^ likelihood ratio statistic for the overall group. Given that we were interested in the relative fit of the above presented different models of change within G1 and G2, we investigated also the contribution offered by each group to the overall χ^2^ value. The idea was to have a more careful indication of the impact of including the latent change factor in a specific group. We also investigated the values of the Comparative Fit Index (CFI), the Tucker Lewis Fit Index (TLI), the Root Mean Square Error of Approximation (RMSEA) with associated 90% confidence intervals, and the Root Mean Square Residuals Standardized (SRMR). We accepted CFI and TLI values >0.90, RMSEA values <0.08, and SRMR <0.08 (see Kline, 2016). Last, we used the Akaike Information Criteria (AIC; Burnham and Anderson, 2004). AIC rewards goodness of fit and includes a penalty that is an increasing function of the number of parameters estimated. Burnham and Anderson (2004) recommend rescaling all the observed AIC values before selecting the best fitting model according to the following formula: Δi = AICi-AICmin, where AICmin is the minimum of the observed AIC values (among competing models). Practical guidelines suggest that a model which differs less than Δi = 2 from the best fitting model (which has Δi = 0) in a specific dataset is said to be “strongly supported by evidence”; if the difference lies between 4 ≤ and ≤ 7 there is considerably less support, whereas models with Δi > 10 have essentially no support.

## Results

We created two parallel forms of the prosociality scale by following the procedure described in Little et al. (2002, p. 166). In Table 1 we reported zero-order correlations, mean, standard deviation, reliability, skewness, and kurtosis for each parallel form. Cronbach's alphas were good (≥0.74), and correlations were all significant at *p* < 0.001. Indices of skewness and kurtosis for each parallel form in both groups did not exceed the value of |0.61|, therefore the univariate distribution of all the eight variables (4 variables for 2 groups) did not show substantial deviations from normal distribution (Curran et al., 1996). In order to check multivariate normality assumptions, we computed the Mardia's two-sided multivariate test of fit for skewness and kurtosis. Given the well-known tendency of this coefficient to easily reject H_0_, we set alpha level at 0.001 (in this regard, see Mecklin and Mundfrom, 2005; Villasenor Alva and Estrada, 2009). Results of Mardia's two-sided multivariate test of fit for skewness and kurtosis showed *p*-value of 0.010 and 0.030 respectively. Therefore, the study variables showed an acceptable, even if not perfect, multivariate normality. Given the modest deviation from the normality assumption we decided to use Maximum Likelihood as the estimation method.

**Table 1:** **Descriptive statistics and zero-order correlations for each group separately (*N* = 250)**.

	**(1)**	**(2)**	**(3)**	**(4)**	***n***
**G1 (INTERVENTION GROUP)**					
(1) Pr1_T1	*0.80*				137
(2) Pr2_T1	0.81	*0.80*			137
(3) Pr1_T2	0.51	0.52	*0.74*		113
(4) Pr2_T2	0.48	0.59	0.78	*0.79*	113
*M*	3.44	3.49	3.62	3.71	−
*SD*	0.75	0.72	0.60	0.62	−
*Sk*	−0.51	−0.60	−0.34	−0.61	−
*Ku*	−0.06	0.43	−0.13	0.02	−
**G2 (CONTROL GROUP)**					
(1) Pr1_T1	*0.77*				113
(2) Pr2_T1	0.76	*0.76*			113
(3) Pr1_T2	0.74	0.67	*0.75*		91
(4) Pr2_T2	0.65	0.73	0.78	*0.75*	91
*M*	3.42	3.49	3.49	3.55	−
*SD*	0.70	0.71	0.65	0.64	−
*Sk*	−0.39	−0.55	−0.27	−0.41	−
*Ku*	−0.12	−0.01	−0.44	−0.49	−

*Pr1_T1, Parallel form 1 of the Prosociality scale at Time 1; Pr2_T1, Parallel form 2 of the Prosociality scale at Time 1; Pr1_T2, Parallel form 1 of the Prosociality scale at Time 2; Pr2_T2, Parallel form 2 of the Prosociality scale at Time 2; M, mean; SD, standard deviation; Sk, skewness; Ku, kurtosis; n, number of subjects for each parallel form in each group*.

*Italicized numbers in diagonal are reliability coefficients (Cronbach's α)*.

*All correlations were significant at p ≤ 0.001*.

### Evaluating the impact of the intervention

**Figure 2:**
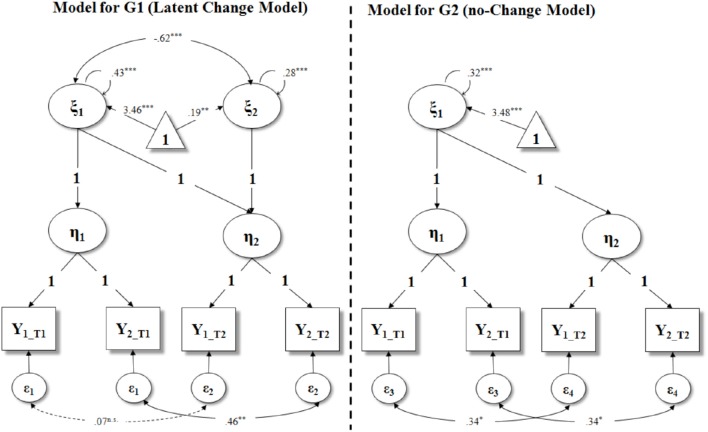
**Best fitting Second Order Multiple Group Latent Curve Model with parameter estimates for both groups**. Parameters in bold were fixed. This model has parallel indicators (i.e., residual variances of observed indicators are equal within the same latent variable, in each group). All the intercepts of the observed indicators (Y) and endogenous latent variables (η) are fixed to 0 (not reported in figure). G1, intervention group; G2, control group; ξ_1_, intercept of prosociality; ξ_2_, slope of prosociality; η_1_, prosociality at T1; η_2_, prosociality at T2; Y, observed indicator of prosociality; ε, residual variance of observed indicator. ^n.s.^ *p* > 0.05; ^*^p < 0.05; ^**^p < 0.01; ^***^p < 0.001.

**Figure 3:**
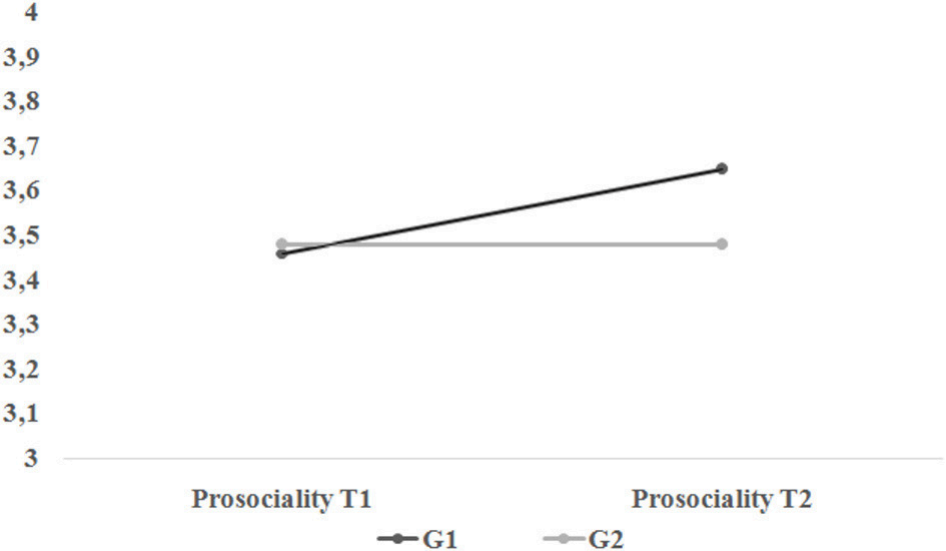
**Trajectories of prosocial behavior for intervention group (G1) and control group (G2) in the best fitting model (Model 2 in Table 2)**.

In Table 2 we reported the fit indexes for the three alternative models (see Appendices B1–B4 for annotated M*plus* syntaxes for each of these). As hypothesized, Model 2 (see also Figure 2) was the best fitting model. Trajectories of Prosociality for intervention and control group separately are plotted in Figure 3. The contribution of each group to overall chi-squared values highlighted how the lack of the slope factor in the intervention group results in a substantial misfit. On the contrary, adding a slope factor to control group did not significantly change the overall fit of the model [$\Delta \chi_{\left(1\right)}^{2}$ = 0.765, *p* = 0.381]. Of interest, the intercept mean and variance were equal across groups (see Table 2, Model 4) suggesting the equivalence of G1 and G2 at T1.

**Table 2:** **Goodness-of-fit indices for the tested models**.

	**NFP**	**χ^2^(*df*)**	**χ^2^G1(*df*)**	**χ^2^G2(*df*)**	**CFI**	**TLI**	**RMSEA [90% CI]**	**SRMR**	**AIC (ΔAIC)**
Model 1 (G1 = A; G2 = A)	16	22.826(12)^*^	18.779(6)^**^	4.047(6)^n.s.^	0.981	0.981	0.085 [0.026,0.138]	0.081	1318.690(9.68)
**Model 2 (G1 = B; G2 = A)**	**17**	**11.143(11)^n.s.^**	**7.096(5)^n.s.^**	**4.047(6)^n.s.^**	**1.00**	**1.00**	**0.010 [0.000,0.095]**	**0.047**	**1309.007(0)**
Model 3 (G1 = B; G2 = B)	18	10.378(10)^n.s.^	7.096(5)^n.s.^	3.282(5)^n.s.^	0.999	0.999	0.017 [0.000,0.099]	0.045	1310.242(1.24)
	**NFP**	**χ^2^(*df*)**	**χ^2^G1(*df*)**	**χ^2^G2(*df*)**	**CFI**	**TLI**	**RMSEA [90% CI]**	**SRMR**	**Δχ^2^(Δ*df*) of M4 vs. M2**
Model 4	15	13.279(13)^n.s.^	7.920(6)^n.s.^	5.359(7)^n.s.^	1.00	1.00	0.013 [0.000,0.090]	0.160	2.136(2)^n.s.^

*G1, intervention group; G2, control group; A, no-change model; B, latent change model; NFP, Number of Free Parameters; df, degrees of freedom; χ^2^G1, contribution of G1 to the overall chi-square value; χ^2^G2, contribution of G2 to the overall chi-square value; CFI, Comparative Fit Index; TLI, Tucker-Lewis Index; RMSEA, Root Mean Square Error of Approximation; CI, confidence intervals; SRMR, Standardized Root Mean Square Residual; AIC, Akaike's Information Criterion*.

*ΔAIC = Difference in AIC between the best fitting model (i.e., Model 2; highlighted in bold) and each model*.

*Model 4 = Model 2 with mean and variance of intercepts constrained to be equal across groups*.

*The full Mplus syntaxes for these models were reported in Appendices*.

n.s. p > 0.05;

* p < 0.05;

** *p < 0.01*.

In Figure 2 we reported all the parameters of the best fitting model, for both groups. The slope factor of intervention group has significant variance (φ_2_ = 0.28, *p* < 0.001) and a positive and significant mean (κ_2_ = 0.19, *p* < 0.01). Accordingly, we investigated the presence of the influence of the initial status on the treatment effect by regressing the slope onto the intercept in the intervention group. Note that this latter model has the same fit of Model 2; however, by implementing a slope instead of a covariance, allows to control the effect of the individuals' initial status on their subsequent change. The significant effect of the intercept (i.e., β = –0.62, *p* < 0.001) on the slope (*R*^2^ = 0.38) indicated that participants who were less prosocial at the beginning increased steeper in their prosociality after the intervention.

## Discussion

Data collected in intervention programs are often limited to two points in time, namely before and after the delivery of the treatment (i.e., pretest and posttest). When analyzing intervention programs with two waves of data, researchers so far have mostly relied on ANOVA-family techniques which are flawed by requiring strong statistical assumptions and assuming that participants are affected in the same fashion by the intervention. Although a general, average effect of the program is often plausible and theoretically sounded, neglecting individual variability in responding to the treatment delivered can lead to partial or incorrect conclusions. In this article, we illustrated how latent variable models can help overcome these issues and provide the researcher with a clear model-building strategy to evaluate intervention programs based on a pretest-posttest design. To this aim, we outlined a sequence of four steps to be followed which correspond to substantive research questions (e.g., efficacy of the intervention, normative development, etc.). In particular, Model 1, Model 2, and Model 3 included a different combinations of no-change and latent change models in both the intervention and control group (see Table 2). These first three models are crucial to identify the best fitting trajectory of the targeted behavior across the two groups. Next, Model 4 was aimed at ascertaining if the intervention and control group were equivalent on their initial status (both in terms of average starting level and inter-individual differences) or if, vice-versa, this similarity assumption should be relaxed.

Importantly, even if the intervention and control group differ in their initial level, this should not prevent the researcher to investigate the presence of moderation effects—such as a treatment-initial status interaction—if this is in line with the researcher's hypotheses. One of the major advantage of the proposed approach, indeed, is the possibility to model the intervention effect as a random latent variable (i.e., the second-order latent slope) characterized by both a mean (i.e., the average change) and a variance (i.e., the degree of variability around the average effect). As already emphasized by Muthén and Curran (1997), a statistically significant variance indicates the presence of systematic individual differences in responding to the intervention program. Accordingly, the latent slope identified in the intervention group can be regressed onto the latent intercept in order to examine if participants with different initial values on the targeted behavior were differently affected by the program. Importantly, the analysis of the interaction effects does not need to be limited to the treatment-initial status interaction but can also include other external variables as moderators (e.g., sex, SES, IQ, behavioral problems, etc.; see Caprara et al., 2014).

To complement our formal presentation of the LCM procedure, we provided a real data example by re-analyzing the efficacy of the YPA, a universal intervention program aimed to promote prosociality in youths (Zuffianò et al., 2012). Our four-step analysis indicated that participants in the intervention group showed a small yet significant increase in their prosociality after 6 months, whereas students in the control group did not show any significant change (see Model 1, Model 2, and Model 3 in Table 2). Furthermore, participants in the intervention and control group did not differ in their initial levels of prosociality (Model 4), thereby ensuring the comparability of the two groups. These results replicated those reported by Zuffianò et al. (2012) and further attested to the effectiveness of the YPA in promoting prosociality among adolescents. Importantly, our results also indicated that there was a significant variability among participants in responding to the YPA program, as indicated by the significant variance of the latent slope. Accordingly, we explored the possibility of a treatment-initial status interaction. The significant prediction of the slope by the intercept indicated that, after 6 months, those participants showing lower initial levels of prosociality were more responsive to the intervention delivered. On the contrary, participants who were already prosocial at the pretest remained overall stable in their high level of prosociality. Although this effect was not hypothesized *a priori*, we can speculate that less prosocial participants were more receptive to the content of the program because they appreciated more than their (prosocial) counterparts the discussion about the importance and benefits of prosociality, topics that, very likely, were relatively new for them. However, it is important to remark that the goal of the YPA was to merely sensitize youth to prosocial and empathic values and not to change their actual behaviors. Accordingly, our findings cannot be interpreted as an increase in prosocial conducts among less prosocial participants. Future studies are needed to examine to what extent the introduction of the YPA in more intensive school-based intervention programs (see Caprara et al., 2014) could represent a further strength to promote concrete prosocial behaviors.

## Limitations and conclusions

Albeit the advantages of the proposed LCM approach, several limitations should be acknowledged. First of all, the use of a second order LCM with two available time points requires that the construct is measured by more than one observed indicators. As such, this technique cannot be used for single-item measures (e.g., Lucas and Donnellan, 2012). Second, as any structural equation model, our SO-MG-LCM makes the strong assumption that the specified model should be true in the population. An assumption that is likely to be violated in empirical studies. Moreover, it requires to be empirically identified, and thus an entire set of constraints that leave aside substantive considerations. Third, in this paper, we restricted our attention to the two parallel indicators case to address the more basic situation that a researcher can encounter in the evaluation of a two time-point intervention. Our aim was indeed to confront researchers with the more restrictive case, in terms of model identification. The case in which only two observed indicators are available is indeed, in our opinion, one of the more intimidating for researchers. Moreover, when a scale is composed of a long set of items or the target construct is a second order-construct loaded by two indicators (e.g., as in the case of psychological resilience; see Alessandri et al., 2012), and the sample size is not optimal (in terms of the ratio estimated parameters/available subjects) it makes sense to conduct measurement invariance test as a preliminary step, “before” testing the intervention effect, and then use the approach described above to be parsimonious and maximize statistical power. In these circumstances, the interest is indeed on estimating the LCM, and the invariance of indicators likely represent a prerequisite. Measurement invariance issues should never be undervalued by researchers. Instead, they should be routinely evaluated in preliminary research phases, and, when it is possible, incorporated in the measurement model specification phase. Finally, although intervention programs with two time points can still offer useful indications, the use of three (and possibly more) points in time provides the researcher with a stronger evidence to assess the actual efficacy of the program at different follow-up. Hence, the methodology described in this paper should be conceived as a support to take the best of pretest-posttest studies and not as an encouragement to collect only two-wave data. Fourth, SEM techniques usually require the use of relatively larger samples compared to classic ANOVA analyses. Therefore, our procedure may not be suited for the evaluation of intervention programs based on small samples. Although several rules of thumb have been proposed in the past for conducting SEM (e.g., *N* > 100), we encourage the use of Monte Carlo simulation studies for accurately planning the minimum sample size before starting the data collection (Bandalos and Leite, 2013; Wolf et al., 2013).

Despite these limitations, we believe that our LCM approach could represent a useful and easy-to-use methodology that should be in the toolbox of psychologists and prevention scientists. Several factors, often uncontrollable, can oblige the researcher to collect data from only two points in time. In front of this (less optimal) scenario, all is not lost and researchers should be aware that more accurate and informative analytical techniques than ANOVA are available to assess intervention programs based on a pretest-posttest design.

## Author contributions

GA proposed the research question for the study and the methodological approach, and the focus and style of the manuscript; he contributed substantially to the conception and revision of the manuscript, and wrote the first drafts of all manuscript sections and incorporated revisions based on the suggestions and feedback from AZ and EP. AZ contributed the empirical data set, described the intervention and part of the discussion section, and critically revised the content of the study. EP conducted analyses and revised the style and structure of the manuscript.

## Funding

The authors thank the students who participated in this study. This research was supported in part by a Research Grant (named: “Progetto di Ateneo”, No. 1081/2016) awarded by Sapienza University of Rome to GA, and by a Mobility Research Grant (No. 4389/2016) awarded by Sapienza University of Rome to EP.

### Conflict of interest statement

The authors declare that the research was conducted in the absence of any commercial or financial relationships that could be construed as a potential conflict of interest.

## Footnotes

^1^Directed by Leder (2000).

^2^Importantly, although classrooms were randomized across the two conditions (i.e., intervention group and control group), the selection of the four classrooms in each school was not random (i.e., each classroom in school X did not have the same probability to participate in the YPA). In detail, participating classrooms were chosen according to the interest in the project showed by the head teachers.
