# Supplementary material for: Evaluating Intervention Programs with a Pretest-Posttest Design: A Structural Equation Modeling Approach
Source: Front Psychol. 2017 Mar 2;8:223. doi: 10.3389/fpsyg.2017.00223 (PMC5332425; doi:10.3389/fpsyg.2017.00223)
Supplement: Supplementary file 1 [file DataSheet1.docx]

**Appendix A**

**Literature search strategies**

**1a)** Enter the following Boolean/Phrase in PsycINFO database:

*AB intervention AND AB pretest AND AB posttest AND AB follow-up*

**1b)** Set the following limiter -> Publication Year: 2006-2016

**2a)** Enter the following Boolean/Phrase in PsycINFO database:

*AB intervention AND AB pretest AND AB posttest NOT AB follow-up*

**2b)** Set the following limiter -> Publication Year: 2006-2016
