# Supplementary material for: Evaluating Intervention Programs with a Pretest-Posttest Design: A Structural Equation Modeling Approach
Source: Front Psychol. 2017 Mar 2;8:223. doi: 10.3389/fpsyg.2017.00223 (PMC5332425; doi:10.3389/fpsyg.2017.00223)
Supplement: Supplementary file 2 [file DataSheet2.docx]

**Appendix B1**

**M*plus* syntax for Model 1 in Table 2.**

Title: Article on two time points;

Model 1 (G1 = no-change G2 = no-change);

Data: file is Frontiers.dat;

Analysis: type is general;

Estimator=ML;

Variable: names are

nord school cond class gender age

PR1_T1 PR2_T1 PR1_T2 PR2_T2;

usevariables are PR1_T1 PR2_T1

PR1_T2 PR2_T2;

missing are all (99);

grouping is cond(1, 2); !(1 = intervention; 2 = control)

Model:

PROS1 by PR1_T1@1 PR2_T1@1;

[PR1_T1@0]; [PR2_T1@0];

PR1_T1; PR2_T1;

PROS2 by PR1_T2@1 PR2_T2@1;

[PR1_T2@0]; [PR2_T2@0];

PR1_T2; PR2_T2;

I by PROS1@1 PROS2@1;

[I]; I;

PROS1;

PROS2;

[PROS1@0];

[PROS2@0];

PR1_T1 with PR1_T2;

PR2_T1 with PR2_T2;

model 1:

PROS1 by PR1_T1@1; PROS1 by PR2_T1@1;

[PR1_T1@0]; [PR2_T1@0];

PR1_T1 (a); PR2_T1 (a);

PROS2 by PR1_T2@1; pros2 by PR2_T2@1;

[PR1_T2@0]; [PR2_T2@0];

PR1_T2 (b); PR2_T2 (b);

I by PROS1@1 PROS2@1;

[I]; I;

PROS1;

PROS2;

[PROS1@0];

[PROS2@0];

PR1_T1 with PR1_T2;

PR2_T1 with PR2_T2;

model 2:

PROS1 by PR1_T1@1; PROS1 by PR2_T1@1;

[PR1_T1@0]; [PR2_T1@0];

PR1_T1 (a1); PR2_T1 (a1);

PROS2 by PR1_T2@1; pros2 by PR2_T2@1;

[PR1_T2@0]; [PR2_T2@0];

PR1_T2 (b1); PR2_T2 (b1);

I by PROS1@1 PROS2@1;

[I]; I;

PROS1;

PROS2;

[PROS1@0];

[PROS2@0];

PR1_T1 with PR1_T2;

PR2_T1 with PR2_T2;

Output: standardized sampstat tech1 mod(3.84);

**Appendix B2**

**M*plus* syntax for Model 2 in Table 2 (the best fitting model).**

Title: Article on two time points;

Model 2 (G1 = latent change G2 = no-change);

Data: file is Frontiers.dat;

Analysis: type is general;

Estimator=ML;

Variable: names are

nord school cond class gender age

PR1_T1 PR2_T1 PR1_T2 PR2_T2;

usevariables are PR1_T1 PR2_T1

PR1_T2 PR2_T2;

missing are all (99);

grouping is cond(1, 2); !(1 = intervention; 2 = control)

Model:

PROS1 by PR1_T1@1 PR2_T1@1; !PROS1 = ETA AT T1

[PR1_T1@0]; [PR2_T1@0];

PR1_T1; PR2_T1;

PROS2 by PR1_T2@1 PR2_T2@1; !PROS2 = ETA AT T2

[PR1_T2@0]; [PR2_T2@0];

PR1_T2; PR2_T2;

I by PROS1@1 PROS2@1; !I = INTERCEPT

[I]; I;

PROS1;

PROS2;

[PROS1@0];

[PROS2@0];

PR1_T1 with PR1_T2;

PR2_T1 with PR2_T2;

s by PROS1 @0; s by PROS2@1; !S = SLOPE

s; [s];

i with s;

model 1:

PROS1 by PR1_T1@1; PROS1 by PR2_T1@1;

[PR1_T1@0]; [PR2_T1@0];

PR1_T1 (a); PR2_T1 (a); !PARALLEL INDICATORS FOR ETA AT T1 (IN G1)

PROS2 by PR1_T2@1; pros2 by PR2_T2@1;

[PR1_T2@0]; [PR2_T2@0];

PR1_T2 (b); PR2_T2 (b); !PARALLEL INDICATORS FOR ETA AT T2 (IN G1)

I by PROS1@1 PROS2@1;

[I]; I;

PROS1@0; !CONSTRAINED TO ZERO

PROS2@0; !CONSTRAINED TO ZERO

[PROS1@0];

[PROS2@0];

PR1_T1 with PR1_T2;

PR2_T1 with PR2_T2;

!!SYNTAX FOR SLOPE

s by PROS1@0; s by PROS2@1;

s; [s];

!!UNCONDITIONAL MODEL (THE ONE REPORTED IN FIGURE 2)

i with s;

!!CONDITIONAL MODEL (CONTROLLING FOR THE INFLUENCE OF INITIAL STATUS+

!!s on i;

model 2:

PROS1 by PR1_T1@1; PROS1 by PR2_T1@1;

[PR1_T1@0]; [PR2_T1@0];

PR1_T1 (a1); PR2_T1 (a1); !PARALLEL INDICATORS FOR ETA AT T1 (IN G2)

PROS2 by PR1_T2@1; pros2 by PR2_T2@1;

[PR1_T2@0]; [PR2_T2@0];

PR1_T2 (b1); PR2_T2 (b1); !PARALLEL INDICATORS FOR ETA AT T2 (IN G2)

I by PROS1@1 PROS2@1;

[I]; I;

PROS1;

PROS2;

[PROS1@0];

[PROS2@0];

PR1_T1 with PR1_T2;

PR2_T1 with PR2_T2;

!!SYNTAX FOR SLOPE (NOTE THAT ALL PARAMETERS ARE CONSTRAINED TO BE ZERO IN THIS GROUP)

s by PROS1@0; s by PROS2@0;

s@0; [s@0];

i with s @0;

Output: standardized sampstat tech1 mod(3.84);

**Appendix B3**

**M*plus* syntax for Model 3 in Table 2.**

Title: Article on two time points;

Model 3 (G1 = latent change G2 = latent change);

Data: file is Frontiers.dat;

Analysis: type is general;

Estimator=ML;

Variable: names are

nord school cond class gender age

PR1_T1 PR2_T1 PR1_T2 PR2_T2;

usevariables are PR1_T1 PR2_T1

PR1_T2 PR2_T2;

missing are all (99);

grouping is cond(1, 2); !(1 = intervention; 2 = control)

Model:

PROS1 by PR1_T1@1 PR2_T1@1;

[PR1_T1@0]; [PR2_T1@0];

PR1_T1; PR2_T1;

PROS2 by PR1_T2@1 PR2_T2@1;

[PR1_T2@0]; [PR2_T2@0];

PR1_T2; PR2_T2;

I by PROS1@1 PROS2@1;

[I]; I;

PROS1;

PROS2;

[PROS1@0];

[PROS2@0];

PR1_T1 with PR1_T2;

PR2_T1 with PR2_T2;

!!ADD SLOPE SYNTAX

s by PROS1 @0; s by PROS2@1;

s; [s];

i with s;

model 1:

PROS1 by PR1_T1@1; PROS1 by PR2_T1@1;

[PR1_T1@0]; [PR2_T1@0];

PR1_T1 (a); PR2_T1 (a);

PROS2 by PR1_T2@1; pros2 by PR2_T2@1;

[PR1_T2@0]; [PR2_T2@0];

PR1_T2 (b); PR2_T2 (b);

I by PROS1@1 PROS2@1;

[I]; I;

PROS1@0; !CONSTRAINED TO ZERO

PROS2@0; !CONSTRAINED TO ZERO

[PROS1@0];

[PROS2@0];

PR1_T1 with PR1_T2;

PR2_T1 with PR2_T2;

!!ADD SLOPE SYNTAX

s by PROS1 @0; s by PROS2@1;

s; [s];

i with s;

model 2:

PROS1 by PR1_T1@1; PROS1 by PR2_T1@1;

[PR1_T1@0]; [PR2_T1@0];

PR1_T1 (a1); PR2_T1 (a1);

PROS2 by PR1_T2@1; pros2 by PR2_T2@1;

[PR1_T2@0]; [PR2_T2@0];

PR1_T2 (b1); PR2_T2 (b1);

I by PROS1@1 PROS2@1;

[I]; I;

PROS1@0; !CONSTRAINED TO ZERO

PROS2@0; !CONSTRAINED TO ZERO

[PROS1@0];

[PROS2@0];

PR1_T1 with PR1_T2;

PR2_T1 with PR2_T2;

!!ADD SLOPE SYNTAX

s by PROS1 @0; s by PROS2@1;

s; [s];

i with s;

Output: standardized sampstat tech1 mod(3.84);

**Appendix B4**

**M*plus* syntax for Model 4 in Table 2.**

Title: Article on two time points;

Model 4 (G1 = latent change G2 = no-change); !like model 2

intercepts are constrained to be equal across groups;

Data: file is Frontiers.dat;

Analysis: type is general;

Estimator=ML;

Variable: names are

nord school cond class gender age

PR1_T1 PR2_T1 PR1_T2 PR2_T2;

usevariables are PR1_T1 PR2_T1

PR1_T2 PR2_T2;

missing are all (99);

grouping is cond(1, 2); !(1 = intervention; 2 = control)

Model:

PROS1 by PR1_T1@1 PR2_T1@1;

[PR1_T1@0]; [PR2_T1@0];

PR1_T1; PR2_T1;

PROS2 by PR1_T2@1 PR2_T2@1;

[PR1_T2@0]; [PR2_T2@0];

PR1_T2; PR2_T2;

I by PROS1@1 PROS2@1;

[I]; I;

PROS1;

PROS2;

[PROS1@0];

[PROS2@0];

PR1_T1 with PR1_T2;

PR2_T1 with PR2_T2;

!!ADD SLOPE SYNTAX

s by PROS1 @0; s by PROS2@1;

s; [s];

i with s;

model 1:

PROS1 by PR1_T1@1; PROS1 by PR2_T1@1;

[PR1_T1@0]; [PR2_T1@0];

PR1_T1 (a); PR2_T1 (a);

PROS2 by PR1_T2@1; pros2 by PR2_T2@1;

[PR1_T2@0]; [PR2_T2@0];

PR1_T2 (b); PR2_T2 (b);

I by PROS1@1 PROS2@1;

[I] (i_mean); I (i_var);

PROS1@0; !CONSTRAINED TO ZERO

PROS2@0; !CONSTRAINED TO ZERO

[PROS1@0];

[PROS2@0];

PR1_T1 with PR1_T2;

PR2_T1 with PR2_T2;

!!ADD SLOPE SYNTAX

s by PROS1 @0; s by PROS2@1;

s; [s];

i with s;

model 2:

PROS1 by PR1_T1@1; PROS1 by PR2_T1@1;

[PR1_T1@0]; [PR2_T1@0];

PR1_T1 (a1); PR2_T1 (a1);

PROS2 by PR1_T2@1; pros2 by PR2_T2@1;

[PR1_T2@0]; [PR2_T2@0];

PR1_T2 (b1); PR2_T2 (b1);

I by PROS1@1 PROS2@1;

[I] (i_mean); I (i_var);

PROS1;

PROS2;

[PROS1@0];

[PROS2@0];

PR1_T1 with PR1_T2;

PR2_T1 with PR2_T2;

!!ADD SLOPE SYNTAX (CONSTRAINED TO ZERO)

s by PROS1 @0; s by PROS2@0;

s@0; [s@0];

i with s @0;

Output: standardized sampstat tech1 mod(3.84);
